# Facial soft tissue changes after nonsurgical rapid maxillary expansion: a systematic review and meta-analysis

**DOI:** 10.1186/s13005-018-0162-8

**Published:** 2018-03-21

**Authors:** Jing Huang, Cui-Ying Li, Jiu-Hui Jiang

**Affiliations:** 1 Department of Orthodontics, Peking University School and Hospital of Stomatology, 22 South Zhongguancun Avenue, Haidian District, Beijing, 100081 China; 2 Central Laboratory, Peking University School and Hospital of Stomatology, 22 South Zhongguancun Avenue, Haidian District, Beijing, 100081 China

**Keywords:** Maxillary expansion, Nasal changes, Soft tissue changes

## Abstract

**Background:**

The present systematic review and meta-analysis aimed to test the hypothesis that no facial soft tissue changes occur after nonsurgical rapid maxillary expansion (RME), in order to provide a reference for orthodontists.

**Methods:**

PubMed, EMBASE, Cochrane Library, OVID, MEDLINE, CINAHL, Scopus, and ScienceDirect databases were electronically and manually searched up to December 2017, and randomized controlled, clinical controlled trials, cohort studies and retrospective studies where soft tissue changes were measured before and after nonsurgical RME were identified. Study appraisal and synthesis were performed by two reviewers who completed the study selection and quality assessment procedures independently and in duplicate. Data from the involved studies were pooled using Revman 5.3.

**Results:**

A total of 1762 articles were identified after the removal of duplicates. After selection and quality assessment, 15 studies met the inclusion criteria for the systematic review, and 13 articles were ultimately included in the meta-analysis. The quality of the involved studies was relatively moderate. Pre-expansion, postexpansion, and postretention data were pooled. The nasal width, alar base width, and distances from the lower lips to the E line showed significant changes after expansion. Moreover, after retention, the nasal width, mouth width, upper philtrum width, and distance from the lower lip to the E line showed significant increases relative to the baseline values. Limitations of the present study included the moderate quality of the included studies and the fact that the results were based on short-term observations of patients in the growth phase.

**Conclusion:**

Our findings suggest that RME results in a significantly increased nasal width, mouth width, upper philtrum width, and distance from the lower lip to the E line after the retention phase. However, the clinical importance of these findings is questionable.

**Electronic supplementary material:**

The online version of this article (10.1186/s13005-018-0162-8) contains supplementary material, which is available to authorized users.

## Background

Rapid maxillary expansion (RME) is routinely adopted by orthodontists to eliminate skeletal maxillary transverse deficiency; it is especially preferred for patients with posterior crossbite, moderate crowding, and sleep-disordered breathing [1–4]. This treatment approach involves the mechanical separation of the midpalatal suture via disruption of the sutural connective tissue by orthopedic forces in a short period of time. This increases the width of the maxillary segments and achieves harmony between the maxillary and mandibular arches [3, 4].

However, Proffit et al. claimed that RME should be cautiously used in preschool-aged children, who are at high risk for developing undesirable nasal morphological changes [5]. Bailey et al. also reported a case involving a 5-year-old girl who underwent RME and developed an unpleasant nasal shape and dorsal hump after 10 days of treatment [6]. Moreover, Haas et al. and Berger et al. suggested that an increase in the soft nasal width is a potential side effect of orthopedic maxillary expansion [2, 7].

One of the primary aims of orthodontists is to improve facial harmony and esthetics while achieving ideal occlusion. Well-balanced facial soft tissue proportions, rather than hard tissue proportions, should be the ultimate aim of orthodontic treatment [8]. Berger et al. initially associated soft tissue alterations with skeletal changes after RME through an analysis of soft tissue changes in patients who underwent orthopedically or surgically assisted RME. They analyzed posteroanterior cephalograms and confirmed that the soft tissue changes/skeletal changes ratio was 1:1 [7]. These findings were supported by those in a recent study by Pangrazio-Kulbersh et al., who used cone beam computed tomography (CBCT) [9].

Although several studies have reported the skeletal and dental effects of RME, only a few studies and scarce data have addressed alterations in the overlying soft tissue. To our knowledge, there is no meta-analysis concerning the effects of RME on facial soft tissues.

The objective of this meta-analysis was to investigate the hypothesis that no facial soft tissue changes occur after nonsurgical RME, in order to provide a reference for orthodontists.

## Methods

This systematic review and meta-analysis followed the Preferred Reporting Items for Systematic Reviews and Meta-Analyses (PRISMA) guidelines. The study was conducted under the ethical guidelines of the 1975 Declaration of Helsinki and was approved by the review committee of the Peking University School and Hospital of Stomatology.

The meta-analysis was designed and conducted according to instructions from the Cochrane Handbook; its study design, participant, intervention, comparison, and outcome definitions were followed.

### Study search

PubMed, EMBASE, Cochrane Library, OVID, MEDLINE, CINAHL, Scopus and ScienceDirect databases were electronically and manually searched up to December 2017. A search strategy was formulated for each database; details are shown in Table 1. Only articles published in English were selected, and those in other languages with no English version available were not considered.

**Table 1:** Search strategies for different databases

Database	Search strategy	Results
Pubmed	((orthodontics[MeSH Terms]) AND ((maxillary expansion) OR palatal expansion technique[MeSH Terms])) AND (face[Title/Abstract] OR mouth[Title/Abstract] OR lip[Title/Abstract] OR nose[Title/Abstract] OR nasal[Title/Abstract] OR naso*[Title/Abstract] OR alar[Title/Abstract] OR soft tissue*[Title/Abstract])	668
Embase	#1 ‘orthodontics’/exp. #2 ‘palatal expansion technique’/exp. #3 ‘maxillary expansion’ #4 ‘soft tissue’:ab,ti #5 face:ab,ti OR mouth:ab,ti OR lip:ab,ti OR nose:ab,ti OR nasal:ab,ti OR naso*:ab,ti OR alar:ab,ti #6 #2 OR #3 #7 #4 OR #5 #8 #1 AND #6 AND #7	282
Crochrane	#1 MeSH descriptor: [Orthodontics] explode all trees #2 MeSH descriptor: [Palatal Expansion Technique] explode all trees #3 face or mouth or lip or nose or nasal or naso* or alar or ‘soft tissue*’:ti,ab,kw (Word variations have been searched) #4 ‘maxillary expansion’ (Word variations have been searched) #5 #2 OR #4 #6 #1 AND #3 AND #5	66
Ovid	1. exp. orthodontics/ 2. exp. palatal expansion technique/ 3. maxillary expansion.af. 4. 2 or 3 5. (face or mouth or lip or nose or nasal or naso* or alar or soft tissue*).af. 6. 1 and 4 and 5	603
MEDLINE Complete (EBSCOhost)	AB (face or mouth or lip or nose or nasal or naso* or alar or ‘soft tissue*’) AND AB orthodontic AND AB ((maxillary expansion) OR (palatal expansion))	154
CINAHL (EBSCOhost)	same as MEDLINE Complete	19
SCOPUS	(TITLE-ABS-KEY(face OR mouth OR lip OR nose OR nasal OR naso* OR alar OR “soft tissue*”) AND TITLE-ABS-KEY(“maxillary expansion” OR “palatal expansion”) AND TITLE-ABS-KEY(orthodontic))	702
Sciencedirect	TITLE-ABSTR-KEY(face OR mouth OR lip OR nose OR nasal OR naso* OR alar OR “soft tissue*”) and TITLE-ABSTR-KEY((orthodontic AND (“maxillary expansion” OR “palatal expansion”)))	73
In total		2567

### Inclusion criteria

The inclusion criteria for the selected articles were as follows: randomized controlled trials (RCTs), clinical controlled trials (CCTs), cohort studies and retrospective studies including human subjects who underwent nonsurgical RME, and the availability of facial tissue measurements obtained before and after RME by direct measurement, two-dimensional (2D) methods, or three-dimensional (3D) methods.

Studies where orthopedic surgery or a surgically assisted technique was used, those where other interventions such as protraction and fixed-bracket therapy were performed during the observational period after RME; those including patients with cleft lip or palate and orthodontic or orthopedic treatment histories; and those categorized as reviews, abstracts, conference papers, case reports, and letters were excluded.

### Selection of studies

Two reviewers (JH and JHJ) completed the study search and selection procedures by screening the titles and abstracts of articles identified via the electronic and manual searches. When the titles and abstracts were insufficient for decision making, we obtained the full text to make a judgment. The full texts of all potential studies were collected for further consideration; the two reviewers independently decided whether to include each article according to the selection criteria. Studies that presented only changes between time periods, with no available data for each time point, were excluded from the meta-analysis. Disagreements were resolved through a discussion among all reviewers.

### Primary and secondary outcomes

The following transversal measurements were collected as the primary outcomes: nasal width (distance between the most lateral points of the left and right soft alar), alar base width (distance between the most lateral points of insertion of the nose into the face), mouth width, and upper philtrum width.

The secondary outcomes included seven sagittal measurements, including the nasal tip prominence, nasolabial angle, upper lip thickness, basic upper lip thickness (superior sulcus to the skeletal A point), soft pogonion thickness, distance from the upper lip to the E line, and distance from the lower lip to the E line. Moreover, four vertical measurements were recorded, including the upper lip height, lower lip height, lower facial height, and height of nose.

### Risk of bias assessment

We compiled and modified a bias assessment scale for this study on the basis of the CONSORT statement. It involved the study design, measurement methods, statistics, and reports to evaluate the value and quality of each included article. As Johnson et al. reported, a sample with 17 per group would have a statistical power of over 80% [10]. For this study, if there was more than one study group, we pooled patients who underwent RME in each article into a total sample. In total, the maximum sum was 17 points; scores of ≥15, scores of < 15 and ≥12 and scores of< 12 were considered to represent high, moderate, and low quality, respectively. Two reviewers (JH and JHJ) independently evaluated the quality of each article; any disagreement was resolved by discussion with the third reviewer (CYL).

### Data extraction and synthesis

Two reviewers (JH and JHJ) separately extracted the relevant data and information. When there were insufficient data in the articles, we contacted the authors by e-mail for additional information.

We pooled the linear and angular changes in certain landmarks, while volumetric analyses and changes in regions were not pooled. Data for more than one RME group were previously synthesized as the sum of the data representing each study.

Statistical analyses were performed using Review Manager 5.3 (The Nordic Cochrane Centre, The Cochrane Collaboration, 2014; Copenhagen, Denmark). Heterogeneity was assessed using the I^2^ statistic with a significance level of α = 0.05. We adopted the mean difference (MD) with the 95% confidence interval (CI). Continuous data were recorded as MDs, while dichotomous data were expressed as relative risks (RRs). Subgroup analyses were conducted on the basis of measurement intervals. Quantitative synthesis would not be conducted if there was high heterogeneity (> 75%). We applied a random-effects model (REM) when there was moderate heterogeneity (50% to 75%); otherwise, when heterogeneity was lower than 50%, a fixed-effects model (FEM) was used.

## Results

### Study selection

**Fig. 1:**
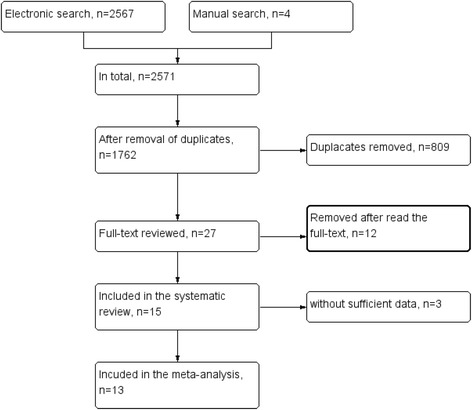
Flow diagram showing the study selection process

The study selection flowchart is depicted in Fig. 1. In total, 2571 articles were identified via electronic and manual searches. After the removal of duplicates, we screened the titles and abstracts of 1762 studies. We obtained the full texts of 27 studies for further consideration. Finally, 15 studies met the inclusion criteria for this systematic review, and 13 were included in the meta-analysis. Articles excluded after reading the full texts had been listed in Additional file 1: Table S1 with reasons explained. We compared the results between reviewers; the interexaminer κ-value was 0.95.

### Bias assessment

We assessed the eligibility and quality of 15 studies and found that five were of high quality and 9 were of moderate quality; one low-quality study was not included in the meta-analysis. The findings of bias assessment are shown in Table 2.

**Table 2:** Quality assessment of the 15 articles included in the systematic review on changes in soft tissues after rapid maxillary expansion

Quality Assessment Criteria(Point)	1	2	3	4	5	6	7	8	9	10	11	12	13	14^a^	15^a^
Age and gender distribution described(1)	1	1	1	1	1	1	1	1	1	1	1	1	1	1	1
Clinical features fully defined(1)	1	1	1	1	1	1	1	1	1	1	0	1	1	1	1
Sample size: adequate(1)	1	1	1	1	1	1	1	1	1	1	1	1	1	0	1
Presence of a blank control(1)	1	0	1	0	0	1	0	1	0	0	1	0	0	0	0
Prospective(1)	0	1	1	0	1	1	1	1	1	1	1	1	1	1	1
Randomization(1)	0	1	1	0	1	0	0	0	1	0	0	0	0	0	0
Appliances described(1)	1	1	1	1	1	1	1	1	1	1	1	1	1	1	1
Interventions fully described(1)	1	1	1	1	1	1	1	0	0	1	1	1	1	1	1
Follow-up defined(1)	0	1	1	1	1	1	0	1	1	1	1	1	0	0	0
Measurement method: appropriate(1)	1	1	1	1	1	1	1	1	1	1	1	1	1	1	1
Assessor blinding(1)	1	0	1	0	0	0	1	0	1	0	1	0	0	0	0
Reliability testing(1)	1	1	1	1	1	1	1	1	1	1	1	1	1	1	1
No dropouts or explained(1)	1	1	1	1	1	1	1	1	1	1	1	1	1	1	1
Statistical analysis: appropriate(1)	1	1	1	1	1	1	1	1	1	1	1	1	1	1	1
Confounders analysed(1)	1	0	1	0	1	1	1	1	1	1	1	1	1	1	1
Results reported: adequate(1)	1	1	1	1	1	1	1	1	1	1	1	1	1	0	0
Reasonable conclusion(1)	1	1	1	1	1	1	1	1	1	1	1	1	1	1	1
Total	14	14	17	12	15	15	14	14	15	14	15	14	13	11	12

^a^articles included in the systematic review but not in the meta-analysis. The number of articles is the same as that in Table 3

### Characteristics of the involved studies

The detailed characteristics of the included studies are summarized in Table 3. The methodological features included size, sex, age, appliance, duration of activation, and retention.

**Table 3:** Details of included articles

No.	Author& Year	Design	Groups	Size	Males/Females	Average Age(year)	Appliance	Expansion duration	Retention duration	Measurement methods	Measurement time
1	Badreddine 2017 [30]	retro- spective	study	20	10/10	8.9 ± 2.16	hyrax expander	3 months	–	CT images	T0,T1
			control	10	5/5	9.2 ± 2.17	–	–	–		
2	Altındis¸ 2016 [23]	RCT	banded RME	14	6/8	12.7 ± 0.6	Hyrax screw	–	3 months	3-D image	T0,T2
			bonded RME	14	7/7	12.4 ± 0.8					
			Modified bonded RME	14	5/9	12.5 ± 0.8					
3	Baysal 2016 [14]	RCT	treated	17	9/8	13.4 ± 1.2	bonded acrylic splint expander	–	6 months	poteroanterior cephalogram and 3-D image	T0,T2
			untreated	17	9/8	12.8 ± 1.3	–	–	–		
4	Torun 2016 [35]	retro- spective	prepubertal	14	10/18	13.91 ± 1.8	Hyrax screw	3–4 weeks	6 months	CBCT and 3-D image	T0,T2
			postpubertal	14							
5	Halıcıoğlu 2016 [31]	RCT	memory-screw	17	9/8	13 ± 1.29	memory- screw	7.76 ± 1.04 days	6.42 ± 0.59 months	lateral cephalograms	T0,T1,T2
			Hyrax-screw	15	8/7	12.58 ± 1.5	Hyrax- screw	35.46 ± 9.39 days	6.17 ± 0.32 months		
6	Uysal 2015 [36]	CCT	study	20	8/12	13.4 ± 0.99	acrylic bonded RME appliance	average 1.1 months	6 months	lateral and anteroposterior radiographs	T0,T1,T2
			control	16	6/10	13.25 ± 1.19	–	–	–		
7	Longo 2014 [34]	cohort	study	28	14/14	12 years 2 months ±3.1 years	banded Hyrax(24 subjects), banded Haas(3), bonded Hyrax(1)	–	–	direct measurement with caliper	T0,T1
8	Santariello 2014 [37]	CCT	study	61	35/26	10.5 ± 1.8	Hyrax type expander	3–4 weeks	nearly 6 months	direct measurement with caliper	T0,T1,T2
			control	41	15/26	10.7 ± 2.2	–	–	–		
9	Pangrazio-Kulbersh 2012 [9]	RCT	banded maxillary expanders	13	7/6	12.6 ± 1.8	banded maxillary expanders	4–6 weeks	6 months	CBCT and 3-D image	T0,T2
			bonded maxillary expanders	10	5/5	13.5 ± 2.1	bonded maxillary expanders				
10	Santos 2012 [22]	cohort	study	20	10/10	9.3 years ± 10 months	modified acrylic Hyrax device	3–4 weeks	6 months	lateral cephalograms	T0,T1,T2
11	Johnson 2010 [10]	CCT	prepubertal	31	12/19	13.1	Hyrax- type expander	average 35 days	average 5.7 months	direct measurement with caliper	T0,T1,T2
			postpubertal	48	17/31						
12	Kilic 2008 [15]	cohort	study	18	3/15	13.5 ± 1.07	rigid acrylic bonded appliance	–	5.95 ± 0.35 months	lateral cephalograms	T0,T1,T2
13	Karaman 2002 [38]	cohort	study	20	10/10	12.8	modified acrylic bonded appliance	5.2 weeks	–	lateral cephalograms	T0,T1
14^a^	Altorkat 2016 [20]	cohort	study	14	7/7	12.6 ± 1.8	Hyrax screw	–	–	3D stereophoto-grammetry	T0,T1
15^a^	Kim 2012 [24]	cohort	study	23	10/13	12.3 ± 2.6	fixed rapid maxillary expander	average 22.8 days	–	CBCT	T0,T1

^a^Articles included in the systematic review but not in the meta-analysis. T0 = pre-expansion, T1 = postexpansion, T2 = postretention

### Data extraction and synthesis

Two reviewers (JH and JHJ) separately extracted and pooled data based on the primary and secondary outcomes. We compared measurements obtained before and after expansion, before expansion and after retention, and after expansion and after retention.

Except distance from upper lip to E line, the I^2^ values for all the other comparisons were < 50%, indicating high homogeneity among groups for most pooled measurements. FEM was used for those comparisons. For upper lip to E line between pre-expansion and postexpansion and between postexpansion and postretention, RME was used.

### Comparisons

Ten baseline and postexpansion measurements (compared in at least two of the included studies), 11 baseline and postretention measurements, and five postexpansion and postretention measurements (to determine the extent of relapse) were compared in forest plots as Additional file 2: Figure S1 and the results are summarized in Table 4A, B, and C, respectively.

**Table 4:** Results of the meta-analysis on changes in soft tissues after rapid maxillary expansion

Outcome	Studies	Subjects	Effect EstimateMD(Fixed, CI 95%)
A.Pre-expansion VS. postexpansion			
Nasal width	5	208	0.84 [0.33, 1.34] ^a^
Alar base width	4	188	0.71 [0.19, 1.23] ^a^
Nasal tip prominence	3	56	0.59 [−0.26, 1.44]
Nasolabial angle	2	52	−0.06 [−4.36, 4.24]
Upper lip thickness	2	38	−0.01 [− 0.82, 0.79]
Basic upper lip thickness	2	38	0.28 [− 0.65, 1.22]
Soft pogonion thickness	2	38	0.01 [−0.79, 0.81]
upper lip to E line	3	72	0.11 [−0.65, 0.88]
Lower lip to E line	3	72	0.75 [0.51, 0.99] ^a^
Height of nose	3	68	1.30 [−0.08, 2.67]
B.Pre-expansion VS. postretention			
Nasal width	6	232	0.87 [0.34, 1.41] ^a^
Alar base width	3	158	0.51 [−0.04, 1.06]
Mouth width	2	59	1.84 [0.66, 3.02] ^a^
Upper philtrum width	2	45	0.74 [0.12, 1.36] ^a^
Nasal tip prominence	4	78	0.26 [−0.99, 1.51]
Nasolabial angle	5	142	−0.88 [−2.96, 1.20]
upper lip to E line	2	52	−0.11 [− 0.33, 0.11]
Lower lip to E line	2	52	0.42 [0.17, 0.66] ^a^
Upper lip height	3	87	−0.38 [−1.17, 0.41]
Lower lip height	2	59	0.48 [−0.47, 1.43]
Lower face height	2	59	0.42 [−1.17, 2.01]
C.Postexpansion VS. postretention			
Nasal width	3	160	−0.13 [−0.70, 0.44]
Alar base width	2	140	−0.20 [− 0.80, 0.39]
Nasal tip prominence	2	38	0.19 [−1.25, 1.63]
upper lip to E line	2	52	−0.25 [−1.27, 0.77]
Lower lip to E line	2	52	−0.34 [− 0.57, − 0.11] ^a^

A. Pre-expansion versus post-expansion; B. Pre-expansion versus postretention; C. Postexpansion versus postretention. ^a^significant

## Discussion

In the present study, we included studies that assessed 3D and 2D images and direct measurements. Scholars have believed that images of the craniofacial complex are more accurate with 3D radiography techniques, which avoid the superimposition and image distortion observed with 2D radiography techniques [11, 12]. However, Weinberg et al. suggested that there was high intraobserver precision among 2D, 3D, and direct measurements, which was supported by the findings in a study by Baysal et al. [13, 14].

A flattened nasal shape and development of a dorsal hump are two of the potential negative effects of RME [15]. According to the present study, the nasal width(MD:0.84 mm, 95% CI:0.33, 1.34) and alar base width(MD: 0.71 mm, 95% CI:0.19, 1.23) significantly increased after active expansion, and nasal width(MD: 0.87 mm, 95% CI:0.34, 1.41) continued to show significant increase during retention. According to previous studies evaluating hard tissues, the skeletal nasal cavity width increased by approximately 2.1–4.5 mm via separation of the lateral walls of nasal cavity after RME [4, 10, 16, 17]. Cameron et al. reported that this change effectively enlarged the nasal volume to facilitate respiration, and it was maintained after 8 years of follow-up [18]. Guyuron suggested that the nasal form was mainly controlled by the nasal frame, and that the shape of the nose was probably changed by alterations in the skeleton [19]. Despite the widening effect, Altorkat et al. found a significant increase in the horizontal nasal tip angle (the left alar-pronasal-right alar angle) [20].

RME is performed to relieve transverse constriction of the maxilla via buccal tipping of the posterior teeth and lateral rotation of the two maxillary halves, which increases the transverse dental and skeletal dimensions [3, 21]. Scholars found that the soft tissue changes after RME were consistent with changes in the underlying hard tissues [7] [9]. In our study, the mouth width(MD: 1.84 mm, 95% CI:0.66, 3.02) significantly increased to a mean of 1.84 mm, with an upper 95% confidence limit of 3.02 mm, which indicated possible clinical importance, particularly in larger populations. Soft tissue stretching is probably the reason for the significant increases in the mouth width and upper philtrum width(MD: 0.74 mm, 95% CI:0.12, 1.36) observed after retention in the present study.

With regard to sagittal measurements, the hard tissue responses after RME are still controversial [4, 16, 22]. Lagravère proved that the maxilla moved downward and forward after RME in a meta-analysis, although the findings were not clinically important [21]. The present study showed no significant sagittal changes in the nasomaxillary region. This was supported by the findings in a report by Altorkat et al. [20]. Moreover, Altındiş et al. claimed that there were no significant changes in the soft facial convexity after RME [23]. This was probably because nose flattening was compensated for by forward movement and growth of the maxilla [15]. In the present study, the distance from the lower lips to the E line(MD: 0.75 mm, 95% CI:0.51, 0.99) showed statistically significant changes after expansion, although the changes did not exceed 1 mm, and significantly relapsed after retention(MD: − 0.34 mm, 95% CI:-0.57, − 0.11), which may be related to movement and rotation of the maxilla and mandible. Transversal stretching of the lips was considered the reason for the significant decrease in the lip thickness reported by Kim et al. [24]; however, our findings revealed no significant changes.

Previous studies have supported the conclusion that RME leads to downward and backward rotation of the mandible [3, 4, 22, 25, 26]. Kiliç et al. found that the H angle was significantly increased, with long-term stability, after RME [15]. This probably represents a favorable effect in patients with Class III malocclusion and an undesirable effect in patients with Class II malocclusion. Scholars have indicated that a bonded expander prevents clockwise rotation of the mandible, thus inhibiting an undesirable increase in the facial height [3, 4, 27–29]. In the present study, we found no significant changes in the height of the lower face, nose, or lips.

However, Badreddine found a significant change in the length of the soft tissue of the nose when they compared the treatment group with the control group [30]. This discrepancy may have occurred because of individual differences between groups, and not as an effect of RME. Thus, we evaluated data obtained before expansion and before retention, rather than spontaneous data for the control group, as control data for quantitative analysis; this was done even when a blank control existed, as observed in a study by Halıcıoğlu et al. [31]. On the basis of our findings, the increase in the height of the nose (MD: 1.30 mm, 95% CI: − 0.08, 2.67) after expansion and elongation of the lower face (MD: 0.42 mm, 95% CI: − 1.17, 2.01) could indicate possible esthetic relevance, particularly in larger populations where an increase of > 2 mm is observed.

The effects of various types or designs of expanders and the sex and age of patients were not evaluated because of the small sample size. Torun et al. claimed that there was no significant difference between prepubertal and postpubertal subjects [32]. This was consistent with the findings of Johnson et al., who stated that the maturation status and sex had no significant effects on the soft tissue changes after RME [10].

All studies included in this systematic review enrolled subjects with an average age of 8 to 14 years who were in the active growth phase. As reported by Quintão et al. and Longo et al., the effects of growth on soft tissues could be eliminated as a variable over a 6-month duration [33, 34]. We presumed that growth would not cause substantial interference with the parameters evaluated during the observational period of up to 6–7 months in all studies included in this meta-analysis. None of the involved studies had a follow-up duration beyond the retention period, because RME was usually followed by fixed-bracket therapy or functional orthodontics. Thus, the results of this study were based on short-term studies and observation, leaving long-term stability open to question. Moreover, these factors are obstacles to future research on changes induced by RME [15].

Because RME is more broadly utilized for adult patients in the current clinical setting, it is crucial to clarify that our findings were based on subjects in the facial skull growth phase, and that the conclusions cannot be extrapolated to the general population. Further studies evaluating soft tissue changes after RME in adults are necessary.

The quality of the articles included in this systematic review was relatively moderate. Only five studies included a blank control group for elimination of the effects of normal growth and development as variables. Randomization was relatively difficult because of ethical problems, and blinding of the assessors was not ensured in over half of the involved studies, which decreased the overall quality. Three of the studies included in the meta-analysis and the two studies included only in the systematic review did not document follow-up data after active expansion; thus, the stability during the retention period remains unknown. Further RCTs with larger samples are necessary to obtain more trustworthy results.

Our findings revealed that most of the evaluated measurements showed a mean change of < 1 mm, which indicated limited clinical or esthetic relevance. In order to provide pertinent and convincing evidence regarding this research question, further investigations with larger samples and appropriate controls are necessary for more accurate evaluation of soft tissue responses after RME and the long-term stability of these changes.

### Limitations

This study is limited by the fact that the results and conclusions were based on patients in the growth phase, and that the observational period was only up to 6 months. Therefore, the findings should be cautiously interpreted with regard to patients outside the growth phase and long-term outcomes.

## Conclusions

Our findings suggest that RME results in a significantly increased nasal width, mouth width, upper philtrum width, and distance from the lower lip to the E line after the retention phase. However, the clinical importance of these findings is questionable.

## Additional files

Additional file 1: **Table S1.** Articles excluded after full text reading and the reasons for exclusion. (DOC 36 kb)

Additional file 2: **Figure S1.** Forest plots of comparisons on changes in soft tissues after rapid maxillary expansion. A. Pre-expansion versus post-expansion; B. Pre-expansion versus postretention; C. Postexpansion versus postretention. (TIFF 1007 kb)

## Abbreviations

2D: two-dimensional
3D: three-dimensional
CBCT: cone beam computed tomography
CCT: clinical controlled trial
CI: confidence interval
CINAHL: Cumulative Index to Nursing and Allied Health Literature
FEM: fixed-effects model
MD: mean difference
PRISMA: Preferred Reporting Items for Systematic Reviews Meta-Analyses
RCT: randomized controlled trial
REM: random-effects model
RME: rapid maxillary expansion
RR: relative risk
