# Supplementary material for: Facial soft tissue changes after nonsurgical rapid maxillary expansion: a systematic review and meta-analysis
Source: Head Face Med. 2018 Mar 21;14:6. doi: 10.1186/s13005-018-0162-8 (PMC5863368; doi:10.1186/s13005-018-0162-8)
Supplement: Supplementary file 1 — Table S1. Articles excluded after full text reading and the reasons for exclusion. (DOC 36 kb) [file 13005_2018_162_MOESM1_ESM.doc]

| **Number** | **Articles excluded after reading full-text** | **Author** | **Year** | **Reasons for exclusion** |
| --- | --- | --- | --- | --- |
| 1 | Treatment Timing for Rapid Maxillary Expansion | Baccetti et al. | 2001 | only hard tissues evaluated |
| 2 | Transverse effects on the nasomaxillary complex one year after rapid maxillary expansion as the only intervention: A controlled study | Baratieri et al. | 2014 | only hard tissues evaluated |
| 3 | Three-dimensional computed tomography analysis of airway volume changes after rapid maxillary expansion | Smith et al. | 2012 | only hard tissues evaluated |
| 4 | Effect of rapid maxillary expansion on skeletal, dental, and nasal structures: a postero-anterior cephalometric study | Cross et al. | 2000 | only hard tissues evaluated |
| 5 | Does the Timing and Method of Rapid Maxillary Expansion Have an Effect on the Changes in Nasal Dimensions? | Basciftci et al. | 2002 | only hard tissues evaluated |
| 6 | A prospective CBCT study of upper airway changes after rapid maxillary expansion | Zeng et al. | 2013 | only hard tissues evaluated |
| 7 | Cephalometric study of alterations induced by maxillary slow expansion in adults | Machado Junior, A. J. et al. | 2006 | only hard tissues evaluated |
| 8 | Transverse effects on the nasomaxillary complex one year after rapid maxillary expansion as the only intervention: a controlled study. | Baratieri, C. L., et al. | 2017 | only hard tissues evaluated |
| 9 | Effects of rapid maxillary expansion on facial soft tissues | Dindaroglu et al. | 2016 | region changes evaluated, not certain landmark |
| 10 | Skeletal, soft tissue, and airway changes following the alternate maxillary expansions and constrictions protocol | Yilmaz et al. | 2015 | not conventional RME |
| 11 | Asymmetric rapid maxillary expansion in true unilateral crossbite malocclusion: A prospective controlled clinical study | Ileri et al. | 2015 | not conventional RME |
| 12 | Photographic assessment of nasal morphology following rapid maxillary expansion in children | da Silva Filho et al. | 2011 | subjective and qualirative analyzed |
| included in systematic review | Immediate effects of rapid maxillary expansion on the naso-maxillary facial soft tissue using 3D stereophotogrammetry | Altorkat et al. | 2016 | only data of changes between time intervals |
| included in systematic review | Evaluation of immediate soft tissue changes after rapid maxillary expansion | Kim et al. | 2012 | only data of changes between time intervals |
