# Supplementary figures and images for: Facial soft tissue changes after nonsurgical rapid maxillary expansion: a systematic review and meta-analysis
Source: Head Face Med. 2018 Mar 21;14:6. doi: 10.1186/s13005-018-0162-8 (PMC5863368; doi:10.1186/s13005-018-0162-8)

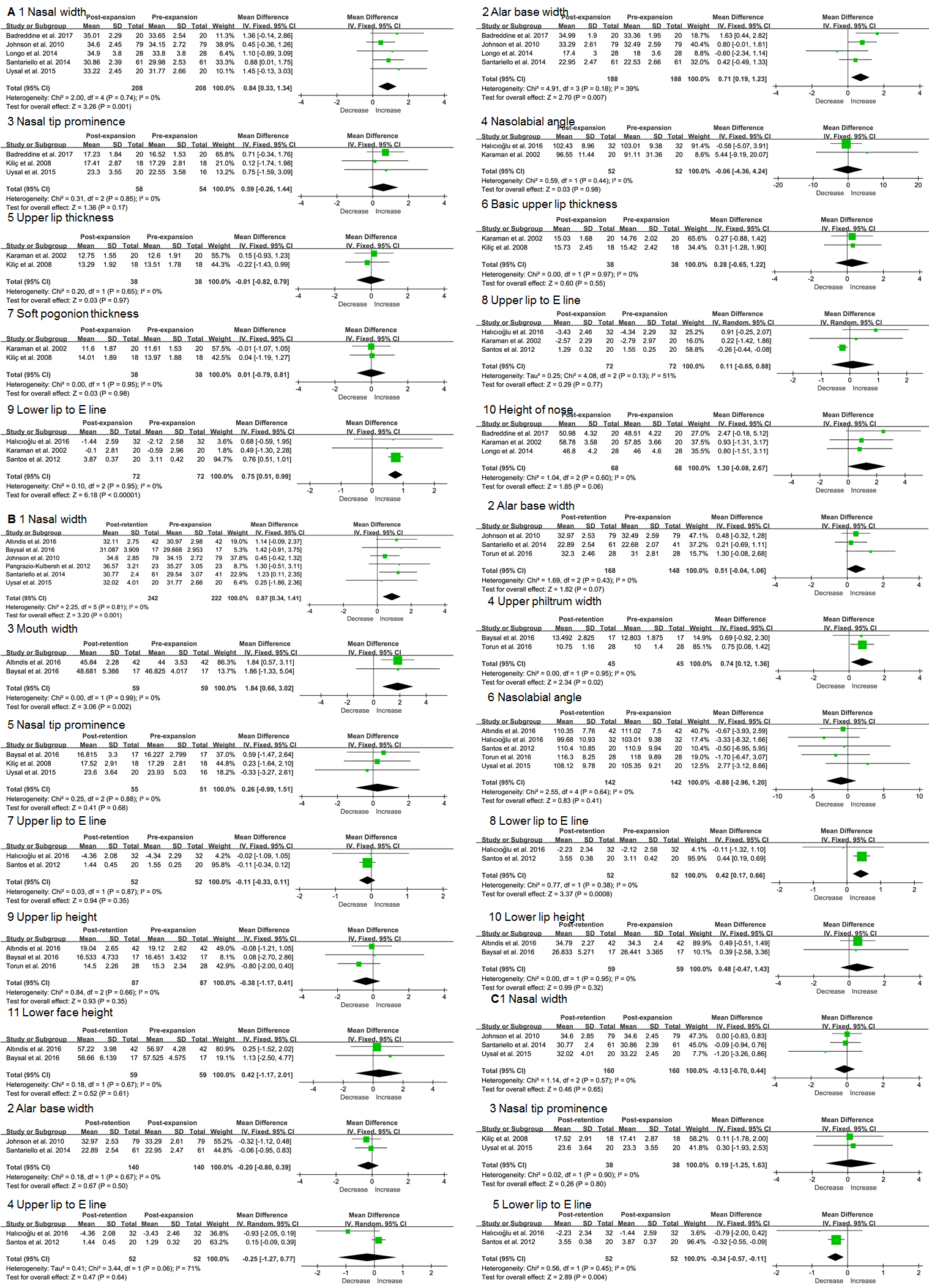

Supplement: Supplementary file 2 — Figure S1. Forest plots of comparisons on changes in soft tissues after rapid maxillary expansion. A. Pre-expansion versus post-expansion; B. Pre-expansion versus postretention; C. Postexpansion versus postretention. (TIFF 1007 kb) [file 13005_2018_162_MOESM2_ESM.tif]
